# Mobile Genetic Elements Drive Antimicrobial Resistance Gene Spread in *Pasteurellaceae* Species

**DOI:** 10.3389/fmicb.2021.773284

**Published:** 2022-01-06

**Authors:** Giarlã Cunha da Silva, Osiel Silva Gonçalves, Jéssica Nogueira Rosa, Kiara Campos França, Janine Thérèse Bossé, Mateus Ferreira Santana, Paul Richard Langford, Denise Mara Soares Bazzolli

**Affiliations:** ^1^Laboratório de Genética Molecular de Bactérias, Departamento de Microbiologia, Instituto de Biotecnologia Aplicada à Agropecuária, Universidade Federal de Viçosa, Viçosa, Brazil; ^2^Grupo de Genômica Evolutiva Microbiana, Laboratório de Genética Molecular de Microrganismos, Departamento de Microbiologia, Instituto de Biotecnologia Aplicada à Agropecuária, Universidade Federal de Viçosa, Viçosa, Brazil; ^3^Section of Paediatrics, Department of Medicine, Imperial College London, London, United Kingdom

**Keywords:** mobile DNA, bacterial resistance, gene transfer, genome evolution, one health

## Abstract

Mobile genetic elements (MGEs) and antimicrobial resistance (AMR) drive important ecological relationships in microbial communities and pathogen-host interaction. In this study, we investigated the resistome-associated mobilome in 345 publicly available *Pasteurellaceae* genomes, a large family of Gram-negative bacteria including major human and animal pathogens. We generated a comprehensive dataset of the mobilome integrated into genomes, including 10,820 insertion sequences, 2,939 prophages, and 43 integrative and conjugative elements. Also, we assessed plasmid sequences of *Pasteurellaceae*. Our findings greatly expand the diversity of MGEs for the family, including a description of novel elements. We discovered that MGEs are comparable and dispersed across species and that they also co-occur in genomes, contributing to the family’s ecology via gene transfer. In addition, we investigated the impact of these elements in the dissemination and shaping of AMR genes. A total of 55 different AMR genes were mapped to 721 locations in the dataset. MGEs are linked with 77.6% of AMR genes discovered, indicating their important involvement in the acquisition and transmission of such genes. This study provides an uncharted view of the *Pasteurellaceae* by demonstrating the global distribution of resistance genes linked with MGEs.

## Introduction

Antimicrobial resistance (AMR), one of the biggest global threats to health and food safety, continues to be driven by misuse of antimicrobials in humans and animals. In bacteria, the acquisition of AMR genes carried on mobile genetic elements (MGEs) can lead to the establishment of multidrug resistance (MDR) (Nikaido, 2009). The collection of all AMR genes is known as the resistome (Wright, 2007) and the entire set of MGEs in a genome defines the mobilome (Siefert, 2009). MGEs are essential in microbial ecology because of their capacity to transfer genes with different roles throughout microbial populations (Rankin et al., 2011).

Horizontal gene transfer (HGT) mechanisms, i.e., conjugation, transduction, transformation, and vesiduction (Soler and Forterre, 2020), mediate dispersion of MGEs, often leading to bacterial evolution, as the introduction of foreign sequences into novel genomic locations can alter phenotypes (Frost et al., 2005; Carr et al., 2021). Larger MGEs can carry AMR genes between microbes (Partridge et al., 2018; Carr et al., 2020), whereas further movement within a host (between co-resident large MGEs and/or chromosomes) can be facilitated by smaller elements (Che et al., 2021). Studies on the resistome-associated mobilome are required to elucidate the dispersion of AMR in bacteria, particularly for human and animal pathogens.

The *Pasteurellaceae* family comprises mainly commensals and pathogens associated with mammalian hosts, including humans and food-production animals (Rosenberg et al., 2013; Michael et al., 2018) where infections have historically been/continue to be treated with antimicrobials, leading to significant problems with AMR (Michael et al., 2018). While MGEs have previously been reported in the *Pasteurellaceae* (Juhas et al., 2007; Moleres et al., 2015; Michael et al., 2018; Szafrański et al., 2019), several aspects of their contribution to MDR have not been addressed. Here, we performed the first large-scale genomic analysis of *Pasteurellaceae* in order to investigate the role of MGEs in the dissemination of AMR genes in this family. We initially focused on the discovery and characterization of MGEs integrated into *Pasteurellaceae* genomes, which provided a substantial dataset to assess their role in the dissemination and shaping of AMR genes in this important family.

## Materials and Methods

### Genome Dataset, Data Processing, and Phylogenetic Analysis

The complete genomes of 345 *Pasteurellaceae* were retrieved from the National Center for Biotechnology Information (NCBI) non-redundant RefSeq database (last accessed in May 2020)^1^ (Supplementary Table 1). Phylogenetic relationships between the genomes were determined using alignment of 16S rRNA sequences using ClustalW (Juraschek et al., 2019), and a maximum likelihood phylogenetic tree was constructed in MEGA X (Kumar et al., 2018), using the Generalized Time Reversible (GTR) and bootstrap confidence value of 1000. The phylogenetic tree was visualized with Interactive Tree of Life (iTOL) (Letunic and Bork, 2019), where the tree was edited and supplemented with genome information. The global distribution of genomes was determined using the ggmap R package version 3.0.0, a heatmap being plotted based on the number of genomes available for each region.

### Identification and Analysis of Insertion Sequences

GenBank format (.gbk) of the genome dataset was used as input for insertion sequence (IS) prediction using ISsaga (Varani et al., 2011) with default parameters. In addition, the recommendations of the Everyman’s Guide to Bacterial Insertion Sequences (Siguier et al., 2015) were used to identify partial elements and provide IS family features.

Hierarchic organization of IS family distribution was visualized in the R environment using the ggplot2 circular package version 3.3.5. ISsaga was used to assess IS ORF genome context. Transposase and adjacent gene sequence were extracted and analyzed for conserved domains using CD-Search (Marchler-Bauer and Bryant, 2004) against the CDD v3.18 database with an expected value threshold of 0.01, and its gene product was inspected for gene ontology annotation through the QuickGO resource at EMBL-EBI (Binns et al., 2009). Next, we created a local database of genes flanking ISs (Supplementary Table 3) and divided them into four classes according to their function: stress response, AMR, adaptation, and virulence. Circular visualization of these classes was created in Circos Table Viewer (Krzywinski et al., 2009).

The genome sequences (.gbk format) of *Mannheimia haemolytica* (strains M42548, USDA-ARS-USMARC-184, and NCTC10643) and *Aggregatibacter actinomycetemcomitans* (strains HK_907, KK1651, and VT1169) were selected as examples of genomes carrying many or few IS copies, and a multiple genome alignment was performed using progressive Mauve (Darling et al., 2004).

### Identification and Analysis of Prophage Elements

We analyzed the prophage elements integrated into the genomes of *Pasteurellaceae* in three critical aspects: prophage-like elements, complete prophages, and novel putative prophage elements. First, we looked for prophage-like elements, i.e., candidate intact prophage that contained phage attachment sites, genes encoding structural phage proteins, genes coding for proteins involved in DNA regulation, insertion into the host genome, and lysis (Arndt et al., 2016; Czajkowski, 2019). We used PHASTER (Arndt et al., 2016) and Prophage Hunter (Song et al., 2019) to predict prophage-like elements. Raw data from the predictions were used for further analysis (Supplementary Tables 4, 5). Next, we screened for complete prophages and prophage-like elements in the classes of intact and active prophage-like elements from PHASTER and Prophage Hunter, respectively. Sequences were subject to BLASTN searches against reference viral genomes already described in the family (Supplementary Table 7) using MegaBLAST (Chen et al., 2015) alignment with a cut-off of 75% of cover and 85% identity, allowing identification of complete prophages found in the bacterial genomes (Supplementary Table 6). More than one complete prophage insertion into the genome was considered a poly-lysogenic event. Finally, we identified novel putative prophages considering the classes of incomplete, questionable, and ambiguous. We also manually inspected the results of mismatched prophage-like elements, according to our criteria, from previous analyses of complete prophages. We screened these sequences for phage-associated functions. Upper boundaries of the novel prophages were determined, wherever possible, by searching for phage integrases from the tyrosine recombinase family at the tRNAs. A sequence identity matrix was built using whole nucleotide sequences of putative novel prophages. These sequences were aligned by Clustal Omega (Sievers and Higgins, 2014) with the default parameters.

To show that putative novel prophages were different from those previously reported in the family (Supplementary Table 7), a synteny analysis using clinker and clustermap.js (Gilchrist and Chooi, 2021) among phages classified by genus was performed. Additionally, we classified a prophage to a family belonging to the order Caudovirales using the occurrence of head-neck-tail module genes detected by the Virfam (Lopes et al., 2014). Phylogenomic analysis of the novel prophages was performed by the ViPTree (Nishimura et al., 2017) webserver. A MegaBLAST analysis against the novel prophages to evaluate their dispersion among *Pasteurellaceae* genomes, using a cutoff of 90% of cover and identity, was carried out. A bipartite network was constructed using vConTACT (Bolduc et al., 2017) to estimate the relationship and clustering of prophages belonging to the *Pasteurellaceae* family.

A graphic representation of novel and previously reported prophages mapped onto the genomes of the family (grouped by genus), based on size, GC content, and number of ORFs was carried out. The annotation of protein sequences was done using GeneMarkS (Besemer et al., 2001) version 4.28 with the sequence type of the phage marked. BLASTP (McGinnis and Madden, 2004) was used to build a local database of the protein sequences, which provided a homologous protein cluster (HPC) with sequences > 30% amino acid identity, > 80% alignment coverage, and clustering *E*-value < 1E-5. Next, the functional annotation of HPC was done using HMMER69 v3.b2 searches with default parameters to the PFAM (Finn et al., 2014). We also analyzed the lifestyle of prophages using the phage Classification Tool Set (PHACTS) (McNair et al., 2012).

### Detection, Delimitation, and Comparative Analysis of Integrative and Conjugative Elements

To identify integrative and conjugative elements (ICEs) in the genomes of the *Pasteurellaceae*, the genomes were inspected for MGE-encoding relaxases, type-IV coupling proteins (T4CP), and the type-IV secretion system (T4SS) gene cluster using the oriTfinder (Li X. et al., 2018) (Supplementary Table 9). ICE sequences experimentally validated for the family were retrieved from the ICEberg (Liu et al., 2018) database version 2.0. Comparative analyses with the ICEberg data allowed the identification of novel putative ICE elements within the *Pasteurellaceae* family (Supplementary Table 11). An element was considered as conjugative when it contained a relaxase, a T4CP, a T4SS gene cluster, and type-specific genes related to mating pair formation (Cury et al., 2017). We inspected attachment sites located between the tRNA and the integrase gene using Repeat Finder plugins on Geneious Prime^®^ version 2020. The opposite boundary of the element was delimited by BLASTN searching, and alignment of the ICE and attachment site sequences using minimum general parameters and filter low complexity region marking. The integrase family was classified by conserved domain searches using CD-Search (Marchler-Bauer and Bryant, 2004) tRNAscan-SE (Chan and Lowe, 2019) and MOBscan (Garcillán-Barcia et al., 2020) were used to identify tRNA genes and classify relaxase families, respectively.

We built a sequence identity matrix using whole nucleotide sequences of putative novel ICEs. These sequences were aligned by Clustal Omega (Sievers and Higgins, 2014) with the default parameters, and a heatmap was generated using the ggplot2 R package. To show that putative novel ICEs were different from those previously reported in the family (Supplementary Table 7), a synteny analysis using clinker and clustermap.js (Gilchrist and Chooi, 2021) among groups of ICEs classified by genus was performed. Next, we performed a global analysis of ICEs mapped onto the genomes, based on size, GC content, and number of ORFs. GeneMarkS (Besemer et al., 2001) was used to annotate predicted protein sequences. A BLASTP alignment to obtain HPC with sequences > 30% protein identity, > 80% alignment coverage, and clustering *E*-value < 1E-5 was done. Next, the functional annotation of HPC was done using HMMER (Wheeler and Eddy, 2013) v3.b2 searches with default parameters to the Pfam (Finn et al., 2014).

Key ICE genes were used for tracking the evolutionary history of these elements. The integrase (*int*), topoisomerase (*parA*), and coupling protein (*traD*) genes were found in almost all ICE sequences. Nucleotide sequences of these genes were aligned using ClustalW to construct maximum likelihood phylogenetic trees. The GTR model and a bootstrap confidence value of 1,000 were applied to each tree. The alignment and phylogenetic analysis were done using MEGA X (Kumar et al., 2018) and edited using iTOL (Letunic and Bork, 2019).

Finally, we constructed a network to evaluate the dispersion of the ICEs among the *Pasteurellaceae* host genomes. A local database with nucleotide sequences of the ICEs was created and MegaBLAST alignments were done, with a cut-off of 75% of cover and identity to consider the interaction between individual ICE and the host genomes, and a tabular representation of this interaction was constructed. The network was visualized using Cytoscape (Shannon et al., 2003).

### Plasmid Dataset and Clustering Plasmid Type

A total of 162 plasmid sequences from the *Pasteurellaceae* were retrieved from the NCBI RefSeq database (last accessed on Nov 2020)^2^ (Supplementary Table 12). We manually curated the plasmid database, eliminating partial plasmid DNA sequences, redundant nomenclature, and unassignable hosts. Relaxases were classified using MOBscan (Garcillán-Barcia et al., 2020), and the nucleotide sequence of *mob* genes was aligned using ClustalW to construct a maximum likelihood phylogenetic tree. The Tamura-Nei (TN) model and a bootstrap confidence value of 1,000 were applied. Alignment and phylogenetic analyses were done using MEGA X and edited using iTOL. To analyze potentially non-mobilizable plasmids, amino acid sequences were aligned using Muscle (Madeira et al., 2019) with the output format adjusted to Phylip sequential. A distant matrix was constructed using the output file through EMBOSS (Madeira et al., 2019). A multi-dimensional graph was created in R. Lastly, to correlate the redundant nomenclature plasmids mentioned above with the One-health concept, an arc diagram was created using the common plasmids found in different species/genomes.

### Resistome Profile Associated With the Mobile Genetic Elements

AMR genes were identified by ResFinder 4.0 (Bortolaia et al., 2020) and the Comprehensive Antibiotic Resistance Database (CARD 2020) (Alcock et al., 2020). The latter was also used to identify any synonymous AMR genes indicated in different published elements using different names. Firstly, we used the context genes associated with AMR provided by ISsaga analysis (Supplementary Table 3) to investigate the association of transposable elements (TEs) with AMR genes. Subsequently, the impact of IS elements in three classes (upstream, downstream, and interruption of AMR gene) according to the transposase position in relation to the AMR genes, was classified. Sequences were extracted from transposons associated with ICEs to analyze the possible transfer of AMR gene sequences. Individual transposases were aligned using the ISfinder database (Siguier et al., 2006), using default parameters (*E*-value ≤ 10-5), and a minimum alignment coverage of 50% and with at least 70% identity was considered. The direct repeat and terminal inverted repeats were manually identified and annotated using Geneious Prime^®^ based BLASTn searches against ISfinder to identify known IS elements.

Fasta sequences of all genomes and MGEs of the *Pasteurellaceae* were used as input for the AMR gene predictions. To show the contribution of the MGEs in carrying AMR genes, we compared the content of AMR genes of these elements to the total number of AMR genes in their respective genomes. For comparison, we used localization of the ICE in the genomes and verified whether the genes were located within the element, and also looked for AMR genes in plasmids belonging to isolates from our genome dataset. Also, individual plasmid and prophage sequences were inspected for AMR genes. The distribution of AMR genes and classes among ICEs and plasmids was represented using the Sankey diagram.^3^ Finally, sequences of the most prevalent AMR genes detected in MGEs were evaluated for selective pressure. AMR gene sequences were aligned in MEGAX (Kumar et al., 2018) and exported in meg format. The program DnaSP v6 (Rozas et al., 2017) was used to carry out Tajima (1989) and Fu and Li (1993) tests.

## Results

### Our Dataset Comprises Highly Diverse and Globally Widespread Genomes

**FIGURE 1:**
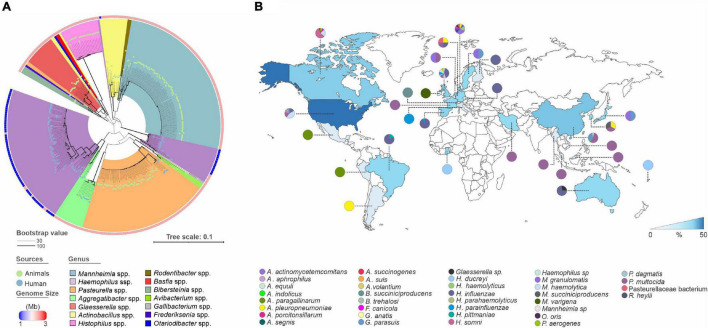
The whole dataset comprises highly diverse and globally widespread genomes. **(A)** Phylogenetic tree of 16S rRNA genes from 345 complete genomes of *Pasteurellaceae* species. From the inside to the outside: the 14 main groups according to genus cluster, the size of the genomes (see legend), and the source of each genome (color dots). The evolutionary history was inferred by using the Maximum Likelihood method based on the General Time Reversible model. The tree is drawn to scale, with branch lengths in the same units as those of the evolutionary distances used to infer the phylogenetic tree. **(B)** Global distribution of publicly available *Pasteurellaceae* genomes by country. The heatmap below the map was plotted based on the number of genomes among the countries. Species distribution by country is represented by pie charts color-code as indicated in the legend.

We surveyed 345 publicly available *Pasteurellaceae* complete genomes, spanning 34 species belonging to 14 genera (Figure 1A). Our genome dataset contains representatives from diverse veterinary species (212), humans (119), and unknown sources (14) (Figure 1A, Supplementary Figure 1, and Supplementary Table 1). Genome sizes ranged from 1.5 Mb in *Haemophilus* spp. to 2.8 Mb in *Mannheimia* spp. (Figure 1A and Supplementary Table 1). We inferred an evolutionary tree using 16S rRNA (Figure 1A). Similar to previous studies (Naushad et al., 2015), our phylogeny indicates that many *Pasteurellaceae* genera have different paraphyletic clades and tend to form different separated clusters, which may reflect misclassifications awaiting resolution. Four different species were assigned to the genus *Mannheimia* (i.e., *Mannheimia granulomatis*, *M. haemolytica*, *Mannheimia varigena*, and *Mannheimia succiniciproducens*), and eight different species have names indicating they are members of the genus *Actinobacillus* [i.e., (*Actinobacillus*) *delphinicola*, *Actinobacillus equuli*, (*Actinobacillus*) *indolicus*, *Actinobacillus lignieresii*, *Actinobacillus pleuropneumoniae*, (*Actinobacillus*) *porcitonsillarum*, (*Actinobacillus*) *succinogenes*, and *Actinobacillus suis*], though square brackets around the genus names indicate species that require reclassification, as they are not *Actinobacillus sensu stricto* (Blackall and Turni, 2020). Our dataset shows a global distribution of sources, though America, Europe and Asia are more highly represented (Figure 1B). *H. influenzae*, *Pasteurella multocida*, *A*. *pleuropneumoniae*, and *A*. *actinomycetemcomitans* had the most genomes available per species, presumably reflecting their importance for human and veterinary health.

### Insertion Sequences Are Broadly Disseminated Affecting Genome Size and Organization

**FIGURE 2:**
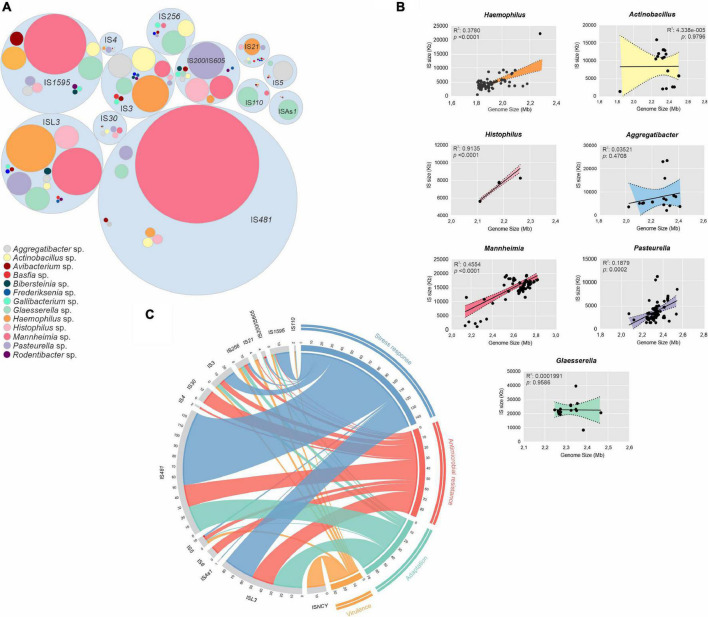
Dissemination, impact in genome size and genetic context of insertion sequences (ISs). **(A)** Hierarchic organization of ISs distribution around the *Pasteurellaceae* species, colored by genus as shown in the legend. **(B)** Correlation graph between genome sizes and ISs grouped by genus. The *x*-axis indicates the genome size and the y-axis indicates the IS size in kilobases. Shaded regions indicate the 95% confidence interval according to the Pearson correlation coefficient. **(C)** Circular visualization of ISs context in four classes according to their flanking genes: Stress response, antimicrobial resistance, adaptation, and virulence (Clockwise direction). Inner connections represent the connection between IS families (anti-clockwise direction) and the function of the flanking genes. Values outside of the ring represent the total number of the IS elements from the respective connection.

We used ISsaga to predict, map and annotate a total of 10,820 ISs, belonging to 19 different families and divided into 12 subgroups (ssgr), in our genome dataset (Supplementary Table 2 and Supplementary Figure 2A). Of the known elements, 37.8% (4,016) were intact and 62.2% (6,611) were partial. The most frequent IS families found were: IS*481*, ISL*3* and IS*1595* ssgr IS*1016* with 2,802 (25.9%), 1,588 (14.67%), 1,211 (11.2%) occurrences, respectively. These elements were broadly disseminated among the *Pasteurellaceae* genomes, with a higher diversity of IS families and an average number of 53, 23, and 94 ISs per genome in *Mannheimia*, *Haemophilus*, and *Glaesserella* genera, respectively (Supplementary Table 2 and Supplementary Figure 2B). Despite the majority of ISs belonging to IS*481*, this family was only found in eight genera, whereas ISL*3*, IS*1595*, and IS*3* were the most widespread within the *Pasteurellaceae* (Figure 2A). Mapping of ISs with regards to genomic locations (Supplementary Table 3) identified 14 IS families inserted within/adjacent to 337 genes associated with stress responses (*n* = 145), AMR (*n* = 89), adaptation (*n* = 42), and virulence (*n* = 61), with IS481 and ISL3 being the most represented families (Figure 2C).

The abundance of IS elements was positively correlated with genome size for *Mannheimia* spp., *Haemophilus* spp., and *Histophilus* spp., but not for *Glaesserella* spp., *Actinobacillus* spp., *Aggregatibacter* spp., and *Pasteurella* spp. (Figure 2B). Alignment of *M*. *haemolytica* genomes (species with many ISs) compared to alignment of *A*. *actinomycetemcomitans* genomes (species with few ISs) revealed, based on synteny analysis, numerous internal rearrangements in genomes possessing a higher number of IS copies compared with those with a lower number of ISs (Supplementary Figure 3), which might have an impact on the genetic organization of these species.

### Comparative Analysis Reveals High Diversity of Prophages in the *Pasteurellaceae*

Using PHASTER and Prophage Hunter, we mapped 2,939 prophage-like elements within our genome dataset (Supplementary Table 4, 5). Of these, 1,398 were classified as intact, questionable, or incomplete by PHASTER, and 1,541 were classified as active or ambiguous using Prophage Hunter (Supplementary Figure 4A). For prophage-like elements classified as intact and active from PHASTER and Prophage Hunter, respectively, we delimited complete prophages comprising eleven different viral species, identified in 193 insertions in 91 *Pasteurellaceae* genomes (Supplementary Table 6 and Supplementary Figure 4B).

**FIGURE 3:**
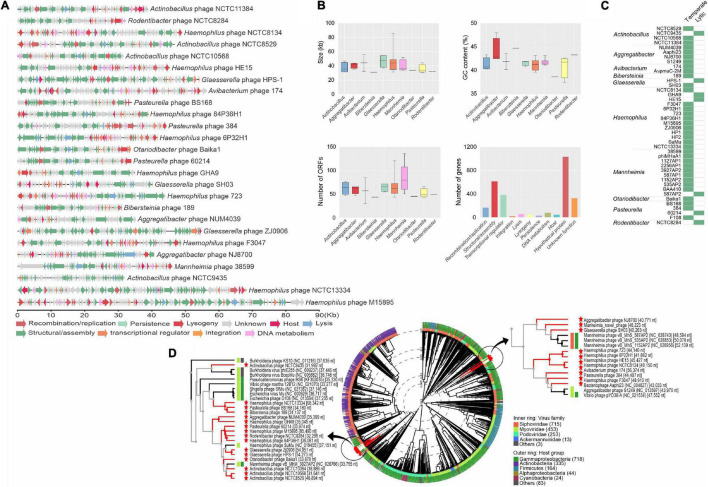
The diversity of prophage elements integrated into *Pasteurellaceae* genomes. **(A)** Representation of novel prophage elements found integrated into *Pasteurellaceae* genomes. Prophage size is indicated in the scale bar (in kilobases) and ORF function is represented by arrows colored below the figure. **(B)** General overview of complete and novel prophages grouped by genus according to phage size (top-left graph), GC content (top-right graph), ORF content (bottom-left graph), and gene function (bottom-right graph). **(C)** Lifestyle classification (temperate or lytic) of complete and novel prophages are shown for all of the prophages in this study. **(D)** Phylogenomic tree analysis of novel prophages and references phages for *Pasteurellaceae* generated on the VipTree website. Colored rings show the families of viruses (inner rings) and host groups (outer rings). The length of the branch is log-scaled. The red stars represent the novel prophages found in this study.

Manual inspection of sequences identified by PHASTER and Prophage Hunter as incomplete/questionable/ambiguous, as well as complete prophages not matching any reported phage for the family, further identified possible novel prophage species, beyond those previously reported in the *Pasteurellaceae* (Supplementary Table 7). This approach identified 23 putative novel prophages in ten different genera (Supplementary Table 8), which exhibit the potential to encode proteins (Open Reading Frames- ORFs) necessary for their assembly and replication (Figure 3A). Whole-sequence alignment and synteny analysis using known *Pasteurellaceae* phages demonstrated that the predicted novel phages are new for this family (Supplementary Figures 4C, 5). These novel phages, predicted to belong to the *Myoviridae* (69%) and *Syphoviridae* (31%) families (Supplementary Table 8), are disseminated in different genomes in our dataset (Supplementary Figure 4D), with the most common being those designated here as *Haemophilus* phage GHA9 and *Mannheimia* phage 38599, and several representing the first report of phage in some of the *Pasteurellaceae* species (Supplementary Figure 4E). In some *M. haemolytica* and *A. actinomycetemcomitans* genomes, poly- insertions were noted (Supplementary Figure 6).

Combining our predicted novel prophage sequences with those previously reported for the *Pasteurellaceae*, overview analysis of the complete dataset revealed that the average size ranged from 31 kb (in *Bibersteinia* spp.) to 47 kb (in *Haemophilus* spp.), with between 30 and 136 ORFs and GC contents of 38–44% (Figure 3B). The average GC contents were similar to those of the genomes (40% ± 2) and the encoded proteins were predicted to be primarily involved in structure/assembly, transcriptional regulation, and recombination/replication, as well as some unknown function (Figure 3B). All prophages belonged to either the *Myoviridae* (73.8%) or *Syphoviridae* (26.2%) family (Supplementary Figure 4F). By comparison to closely related prophages, 83.3% are predicted to be temperate and 16.7% lytic (Figure 3C). Phylogenomic analysis revealed two distant clades, both containing monophyletic groups. None of the putative novel prophages grouped with previously reported *Pasteurellaceae* phages (cluster with 100% identity), confirming the novelty of our findings (Figure 3D). Results of VConTACT analysis further support the viral relationships among the published *Pasteurellaceae* phages, indicating four different clusters (according to the viral family), with homogeneous groups, found for *Aggregatibacter* and *Mannheimia*, and heterogeneous groups for *Haemophilus*, *Mannheimia*, and *Pasteurella* (Supplementary Figure 6). These results agree with the phylogenomic analysis, suggesting a strong evolutionary relationship amongst *Pasteurellaceae* phages.

### *Pasteurellaceae* Genomes Contain Heterogeneous Groups of Disseminated Integrative and Conjugative Elements

**FIGURE 4:**
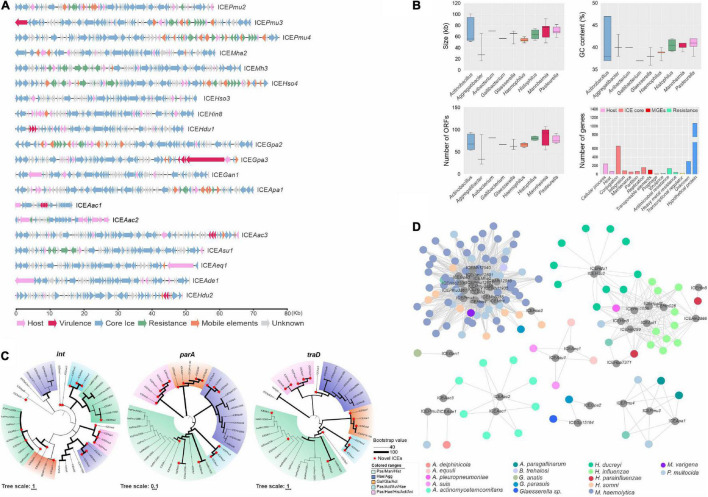
Heterogeneous groups of integrative and conjugative elements (ICEs). **(A)** Representation of novel ICEs found integrated into *Pasteurellaceae* genomes. ICE size is indicated in the scale bar (in kilobases) and ORF functions are represented by arrows colored below the figure. **(B)** General overview of reported and novel ICEs of the family grouped by genus according to ICE size (top-left graph), GC content (top-right graph), ORF content (bottom-left graph), and gene function (bottom-right graph). **(C)** The evolutionary history of integrase (*int*), topoisomerase (*parA*), and coupling protein (*traD*) genes from ICEs inferred by using the Maximum Likelihood method based on the General Time Reversible model. The tree is drawn to scale, with branch lengths in the same units as those of the evolutionary distances used to infer the phylogenetic tree. Colored clusters represent conserved species groups among the trees. Bootstrap values are represented by the line thickness (see legend in the figure). **(D)** Network dispersion analysis of the ICEs among the *Pasteurellaceae* species (host genomes) represented in the species group. ICEs are displayed in dark gray circles with respective names.

OriTFinder analysis identified possible ICEs in our dataset. Results indicated several regions with high potential for self-transferability in 126 genomes comprising ten genera (Supplementary Table 9 and Supplementary Figure 8A). Using ICEfinder and MegaBLAST against known *Pasteurellaceae* ICEs (Supplementary Table 10), we identified nine previously reported ICEs in the genomes. We additionally found evidence of 20 putative novel ICEs (Supplementary Table 11), which exhibit typical modular structures, with blocs of core genes related to ICE replication and dissemination interspersed with cargo genes encoding functions that may benefit the host bacterium (such as resistance or virulence-related genes) (Figure 4A). Most ICEs are preferentially integrated into tRNA sites and encode an integrase belonging to the Xer family and a MOB_H_ relaxase (Supplementary Table 11 and Supplementary Figure 8B). Whole sequence alignment and synteny analysis with previously reported *Pasteurellaceae* ICEs demonstrated the novelty of the 20 putative ICEs (Supplementary Figures 8C, 9).

Collective analysis of the previously reported and novel ICEs indicated that they ranged from 16 to 78 kb, encoding 23–107 ORFs with GC contents from 37 to 41% (averaging 40%, similar to the genomes). Most of the encoded proteins have predicted functions related to ICE replication/dissemination, as well as antimicrobial and/or heavy-metal resistance, however, few virulence genes were found (Figure 4B). Using the key genes *int*, *parA*, and *traD* (where present), we generated phylogenies to determine evolutionary relationships between the various ICE and identified conserved clusters within some genera which encompasses species that may cohabit in the host, such as *Pasteurella* spp., *Mannheimia* spp. and *Histophilus* spp., and other groups in *Haemophilus* spp. and *Aggregatibacter* spp. (Figure 4C). The *parA* gene was the most informative for tracing the evolutionary history of these ICEs. Results also indicate the presence of putative novel ICEs in seven of the *Pasteurellaceae* species (Supplementary Figure 8D).

Network analysis revealed that some related ICEs found in the *Pasteurellaceae* are heterogeneous and widespread among species, e.g., the cluster of ICEs (not yet assigned an ICE family designation) found in *M*. *haemolytica*, *P*. *multocida*, and *H*. *somni*, and the ICE*Hin1056* family of ICEs found in *H. influenzae*, *Haemophilus parainfluenzae*, *A. pleuropneumoniae*, and *Haemophilus ducreyi*. By contrast, some ICEs were found exclusively in *A. actinomycetemcomitans* genomes and do not connect with any other group (Figure 4D). In several *H. ducreyi* isolates (GHA3, GHA5, GHA8, and GHA9), two coexisting ICEs (ICE*Hdu2* and ICE*Hin1056*) were identified (data not shown).

### Plasmids Are a Miscellaneous Class of Mobile Genetic Element in *Pasteurellaceae*

**FIGURE 5:**
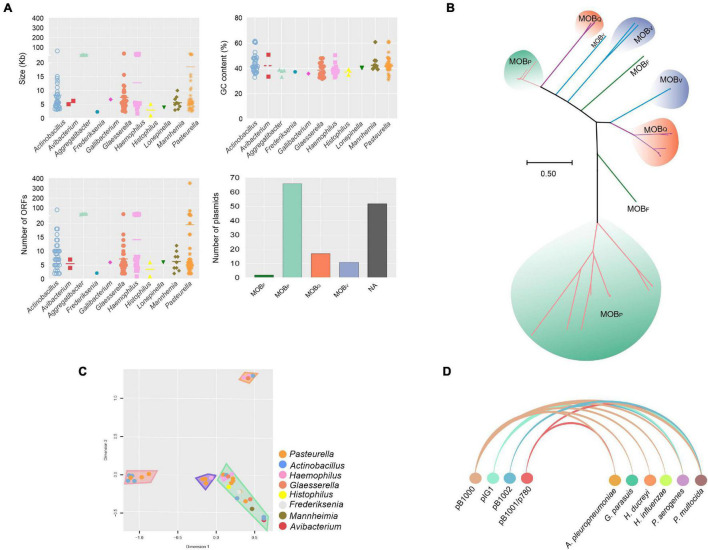
General characteristics and clustering of plasmids found in the *Pasteurellaceae* family. **(A)** A general overview of plasmids in the *Pasteurellaceae* family grouped by genera according to plasmid size (top-left graph), GC content (top-right graph), ORF content (bottom-left graph), and relaxase family (bottom-right graph). **(B)** Evolutionary history of *mob* genes from plasmids inferred by using the Maximum Likelihood method based on the General Time Reversible model. The tree is drawn to scale, with branch lengths measured in the number of substitutions per site. **(C)** A multidimensional graph for analysis of the potentially non-mobilizable plasmid by sequence comparison. Clusters are highlighted in the graph in color-codes that represent the taxonomic group present in that taxon of plasmids. **(D)** Arc diagram representing four plasmids identified in different species/genomes, pB1000, pIG1, pB1002, and pB1001/pB780 (there is no deposit of the plasmid pB1000 identified in *A. pleuropneumoniae*, however, this has already been previously reported (Bossé et al., 2017).

Plasmids are extrachromosomal MGEs that contribute significantly to AMR gene dissemination by HGT (Rodríguez-Beltrán et al., 2021). For our analysis, we retrieved 162 complete *Pasteurellaceae* plasmid sequences from the NCBI RefSeq database (Supplementary Table 12), comprising plasmids from worldwide representatives of 22 species, with *Glaesserella parasuis, P. multocida*, and *A. pleuropneumoniae* having the most entries (Supplementary Figure 10). Although all available plasmids are listed in Supplementary Table 12, where different accession numbers exist for the same plasmid, only one representative sequence of each was included in our analysis to avoid bias. Of the 151 different plasmids analyzed, the smallest was 1 kb and the largest 325 kb. The number of ORFs ranged from 1 to 353, with GC contents from 31 to 61% (average of 41%). Using MOBscan, plasmids were classified according to the type of relaxase encoded, with most assigned to the MOB_P_, MOB_Q_, MOB_V_, and MOB_F_ families (Figure 5A), whereas 52 were designated as potentially non-mobilizable. Results of phylogenetic analysis of the different MOB genes revealed that, even within families, there are polyphyletic clusters, indicating the wide diversity of these sequences (Smillie et al., 2010; Figure 5B and Supplementary Figure 11). Whole-sequence alignments of the potentially non-mobilizable plasmids revealed four clusters of related, but not identical, plasmids present in multiple species (Figure 5C), possibly indicating host-specific divergence following the loss of mobilization function. Notably, four particular plasmids were present in multiple *Pasteurellaceae* species known to infect different animal hosts, i.e., pIG1 (Wright et al., 1997), pB1002 (San Millan et al., 2010), pB1001/p780 (San Millan et al., 2010; Li Y. et al., 2018), and pB1000 (San Millan et al., 2010; Gangaiah et al., 2016; Bossé et al., 2017), the last of which is found in five species including the human pathogens *H. influenzae* and *H. ducreyi*, indicating a One Health concern (Figure 5D).

### The Role of Mobile Genetic Elements in the Dissemination and Acquisition of Antimicrobial Resistance Genes in the *Pasteurellaceae*

Comprehensive analysis of all *Pasteurellaceae* chromosome and plasmid sequences in our datasets revealed a total of 33 different AMR genes were mapped to 478 locations onto 131 genomes from 10 genera (Supplementary Figure 12A). Genes encoding resistance to aminoglycosides, tetracyclines, and sulfonamides were the most represented classes identified (Supplementary Figure 12B). Multiple AMR genes were found in many genomes, and 371 of the 478 AMR genes were associated with MGEs (Supplementary Figure 12C). Also, a wide diversity of AMR genes has been found in species from animal reservoirs such as *P. multocida*, *M. haemolytica* and *Bibersteinia trehalosi*. However, the same was not observed for human pathogens.

**FIGURE 6:**
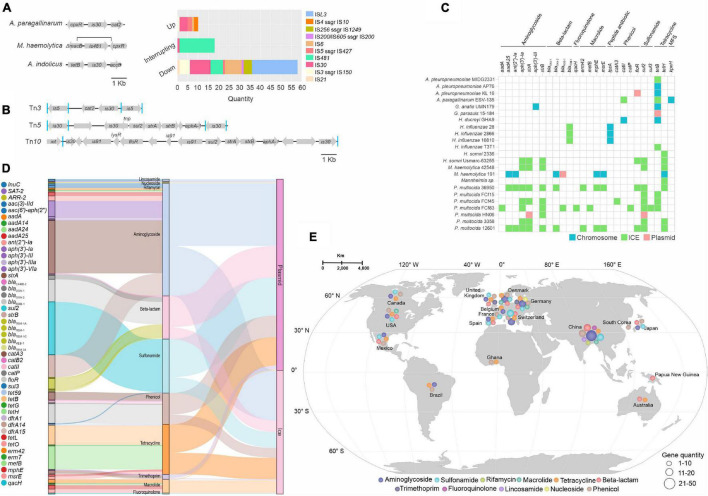
Mobile genetic elements (MGEs) role in dissemination of antimicrobial resistance (AMR) genes. **(A)** Three representative ISs and AMR gene contexts found in this study. Figure highlights, in order, ISs from three different species (genomes) upstream, interrupting, and downstream of AMR genes. The bar graph (top-left graph) shows the values from the previous context for ten IS families. **(B)** Representation of the Tn*3*, Tn*5*, and Tn*10* transposon families carrying AMR genes. Squares in blue identify direct repeats of the IS elements. The name of the AMR gene is shown inside the arrows. **(C)** Comparison of MGEs (green and pink) and their host genomes (in blue) carrying AMR genes. The name of AMR genes and their classes are shown at the top of the table. **(D)** Sankey diagram representing the diversity of AMR genes and their classes; the distribution of these genes between plasmids and ICEs. **(E)** Geographic distribution of AMR classes found within MGEs, color-coded as to their AMR classes. The scale below the map indicates the quantity of AMR genes found for each class.

Determination of TE genetic context with regards position upstream, downstream, or interrupting ORFs mediating AMR (using the dataset shown in Figure 2C and Supplementary Table 3), we identified ten IS families (IS*L3*, IS*481* and IS*30* being most prevalent) mapping to such sites (Figure 6A. In some *M. haemolytica* genomes, IS*481* interrupts and potentially impacts the macrolide-resistance gene *macB* and the fluoroquinolone (norfloxacin and enoxacin) resistance gene *mdtH*. IS*30* in *B. trehalose* interrupts *marB*, a repressor of the *marRAB* operon involved in activation of AMR and oxidative stress genes (Ariza et al., 1994; Figure 6A). Members of the Tn*3*, Tn*5*, and Tn*10* transposon families were mapped to ICEs carrying sulfonamide, phenicol, and aminoglycoside resistance genes (Figure 6B).

Although we found prophage-like elements carrying AMR genes in the genomes of *H. influenzae* and *A. indolicus*, no AMR genes were found in our dataset of complete prophages (Supplementary Table 13). In contrast, a total of 33 different AMR genes were mapped to 195 locations onto 103 plasmids (68% of the plasmids), and a total of 26 different AMR genes were mapped to 126 locations onto 28 ICEs (65% of the ICEs), including ICEs disseminated among different species of the family, with genes encoding resistance to 10 and 8 classes of antimicrobials, respectively (Supplementary Figures 13A,B and Supplementary Table 13). In plasmids, genes for resistance to aminoglycosides and sulfonamides were most prevalent, whereas genes for resistance to aminoglycosides and tetracyclines were the most common in ICEs (Supplementary Table 13). We assessed the contribution of the ICEs and plasmids carrying AMR genes compared to their respective chromosome, and we found that the majority of AMR genes are exclusively associated with ICEs (Figure 6C).

Some classes of AMR genes were exclusively associated with specific elements, e.g., genes for resistance to rifamycin, lincosamide, and N-glycosides were only found in plasmids, and fluoroquinolone resistance genes only in ICEs (Figure 6D). But most classes of AMR genes were distributed among both ICEs and plasmids (Figure 6D) and several plasmids and ICEs encoded multiple AMR genes (Supplementary Figure 13C).

Copies of the chromosomally encoded *kpnH* gene, mediating resistance to various antimicrobials such as azithromycin, ertapenem, imipenem, norfloxacin, and polymyxin B (colistin) (Srinivasan et al., 2014), found in human and animal pathogens share 75% nucleotide identity. Whereas, some plasmid and ICE-encoded resistance genes, such as *tetB*, *catII*, and *bla*_*ROB*–1_, show > 99% nucleotide identity between human and animal pathogens. However, only the pB1000 plasmid (carrying bla_ROB–1_) shows clear evidence of transfer, with identical sequences found in pathogens from five different host species, including humans (Figure 5D). In contrast, many AMR genes were found exclusively in human pathogens (e.g., *hmrM*, *catS*, *lpsA*, and *bla*_*TEM*–1_) or animal pathogens (e.g., *floR*, *strA*, *strB dfrA*, *ermF*, and *bla*_*OXA*_), despite being MGE-associated. In total, 87.24% (*n* = 629) of AMR genes were found in animal and 12.76% (*n* = 92) in human pathogens.

Some AMR genes associated with MGEs were more globally distributed than others in our dataset, e.g., the *bla*_*ROB*–1_, *floR*, *strA*, *sul2*, and *tetB* genes were more abundant in isolates from Europe and Asia (Figure 6E). In contrast, 28 AMR genes were associated with specific localities (Supplementary Figure 13D). Neutrality analysis of the most prevalent MGE-associated AMR genes per geographic location indicated possible recent selection for resistance to sulfonamides (*sul2*) in Europe (Tajima’s D –2.54153, *p* < 0,001; Fu and Li D*: –4.3437, *p* < 0.02; Fu and Li’s F* test statistic: –4.43062, *p* < 0.02) and tetracyclines (*tetB*) in Asia (Tajima’s D: –1.27745; Fu and Li’s D*: –1.27336, *P* < 0.02; Fu and Li’s F* test statistic: –1.38944, *P* < 0.02).

## Discussion

The *Pasteurellaceae* family is composed of a diverse group of Gram-negative bacteria comprising commensals and pathogens of human and animal hosts. These bacteria are part of the normal microbiota of several animals, including humans (Wang et al., 2010; Wilson and Ho, 2013) and can mediate HGT via conjugative mobile elements, with some species also competent for natural transformation (Sinha et al., 2008). The dissemination of microbial MDR determinants represents an increasingly significant problem. However, most efforts are focused on understanding the epidemiology of MDR strains rather than mechanisms of dissemination of the AMR genes by MGEs.

In our investigation of the mobilome, we discovered a vast repertoire of MGEs in *Pasteurellaceae*, some of which were quite similar and distributed across taxa. Our analyses also indicated that the co-occurrence of several types of MGEs (prophages, ICEs, and ISs) is frequent, as shown in some *M. haemolytica*, *H. ducreyi*, *H. influenzae*, *A. actinomycetemcomitans*, and *Avibacterium paragallinarum* strains. All of these discoveries, based on the existence of key genes carried by these elements, highlight the relevance of MGEs in the evolution, diversity, and ecology of *Pasteurellaceae*. By systematically investigating the resistome-associated mobilome in publicly available complete *Pasteurellaceae* genomes and plasmids, our study reveals the importance of MGEs in the dispersion of AMR genes within this family. We recognize that due to the type of genomes considered, we did not capture the whole diversity of MGEs and AMR genes, though many efforts have shown the limitation of the characterization/discovery of MGEs in draft genomes (Gonçalves et al., 2020a,b). However, we have presented the most comprehensive and curated dataset of the mobilome found within 345 complete genomes of the *Pasteurellaceae*, comprised of 10,820 IS elements, 43 complete prophages, 43 ICEs, and 162 plasmid sequences. Our findings expand the diversity of MGEs for the family, including the first report of prophages and ICEs for some species. We identified diverse groups of MGEs, both adapted to the host bacterial genome and highly disseminated throughout the family. Importantly, we demonstrated the role of ICEs, plasmids, prophages, and transposons in the dissemination of AMR genes in members of the *Pasteurellaceae* family.

The occurrence of AMR and in some cases MDR has been documented in the *Pasteurellaceae* (Archambault et al., 2011; Woolums et al., 2018; Van Driessche et al., 2020) but the association of AMR genes with MGEs has received comparatively little attention. Despite suitable management, including biosecurity and vaccines, the use of antimicrobial agents to control infections caused by pathogenic *Pasteurellaceae* is widespread (Michael et al., 2018). However, the emergence of multidrug-resistant bacteria increasingly makes the use of antimicrobials problematic. We discovered that most AMR genes found in *Pasteurellaceae* genomes are associated with ICEs and plasmids since few AMR genes were found in genomes lacking these MGEs. Furthermore, most *Pasteurellaceae* ICEs and plasmids have one or more AMR genes, and these MGEs are, in most cases, responsible for the observed MDR phenotypes, which have a direct influence on the fitness of the species in this family in their environment.

Antimicrobial resistance might be regarded as a colonization factor in the presence of drugs. The dissemination of AMR genes via MGEs in *Pasteurellaceae* might be interpreted as a mechanism by bacterial species to stand out in a microbial community and become more effective during host colonization (Martínez and Baquero, 2002). The repertoire of AMR gene found in the *Pasteurellaceae* mediates resistance to highly important antimicrobials for human and animal treatments (aminoglycosides, beta-lactams, phenicols, sulfonamides, tetracyclines, and lincosamides) (OIE, 2017; WHO, 2019). The gene diversity in pathogens from animal reservoirs was higher than that seen in human pathogens, which is consistent with the dispersion of MGEs among bacterial species. Although our results have shown that most AMR genes are associated with MGEs, dissemination of these elements from veterinary to human pathogens, a concern that is directly related to the One Health concept (Hernando-Amado et al., 2019), does not appear to be common within the *Pasteurellaceae*, although our analyzes have shown that some ICEs are widely disseminated among *Pasteurellaceae* species. One example, noted previously by others (Alvaro et al., 2009; San Millan et al., 2010; Bossé et al., 2017) and seen again in this study, is for the beta-lactam resistance plasmid, pB1000. This plasmid has been identified in both human and veterinary *Pasteurellaceae* pathogens, including *P. multocida*, one of the few members of this family capable of infecting a range of animals, including humans. It should be noted that our results do not preclude the possibility of transfer of MGE-associated AMR determinants from members of the *Pasteurellaceae* to other co-resident bacterial species found within the same animal host, some of which may be zoonotic and therefore present a greater threat to human health (Köck et al., 2021).

The use of genomic data to track global AMR has revolutionized diagnostic microbiology, mostly due to improvements in sequencing technologies and increasing numbers of publicly available genomes, providing an opportunity to expand and align with a One Health surveillance framework (Hendriksen et al., 2019). For AMR surveillance purposes, draft genome sequences have been shown to be sufficient for the identification of genes conferring resistance to a number of antimicrobials, including for members of the *Pasteurellaceae* (Bossé et al., 2017). However, short-read sequencing is not capable of assembling across repeat regions, common in MGEs, and does not give an overall picture of the abundance of these elements within genomes.

## Conclusion

In conclusion, the MGEs described in this study reveal the significance of these elements in the ecology of *Pasteurellaceae* species, mostly regarding the dissemination of AMR genes. To our knowledge, the resistome-associated mobilome data generated in our study represents the most comprehensive description of MGE-associated AMR genes for the *Pasteurellaceae*, providing a valuable resource for future research. Such knowledge is critical for the effective design and interpretation of experimental data to elucidate mechanisms of AMR and to facilitate the development of effective strategies to control resistant bacteria. Lastly, we reinforce that similar approaches, as employed here, can be used to inform future decisions toward the surveillance of AMR genes and to gain insights into their microbial ecology and evolution.

## Data Availability Statement

The datasets presented in this study can be found in online repositories. The names of the repository/repositories and accession number(s) can be found in the Supplementary Material for this manuscript which is online available at: https://doi.org/10.6084/m9.figshare.c.5538240.v2.

## Author Contributions

GCS, OSG, JNR, KCF, JTB, MFS, PRL, and DMSB conceived the study. GCS, OSG, JNR, and KCF provided the data. GCS, OSG, JNR, KCF, JTB, and MFS analyzed the data. GCS, OSG, MFS, JB, PRL, and DMSB wrote the manuscript. All authors contributed to the article and approved the submitted version.

## Conflict of Interest

The authors declare that the research was conducted in the absence of any commercial or financial relationships that could be construed as a potential conflict of interest.

## Publisher’s Note

All claims expressed in this article are solely those of the authors and do not necessarily represent those of their affiliated organizations, or those of the publisher, the editors and the reviewers. Any product that may be evaluated in this article, or claim that may be made by its manufacturer, is not guaranteed or endorsed by the publisher.

## Footnotes

1
ftp.ncbi.nlm.nih.gov/genomes/refseq/

2
https://ftp.ncbi.nih.gov/refseq/release/plasmid/

3
http://sankeymatic.com/
