# Supplementary material for: Mobile Genetic Elements Drive Antimicrobial Resistance Gene Spread in Pasteurellaceae Species
Source: Front Microbiol. 2022 Jan 6;12:773284. doi: 10.3389/fmicb.2021.773284 (PMC8777487; doi:10.3389/fmicb.2021.773284)
Supplement: Supplementary file 1 [file Data_Sheet_1.zip › Supplementary Figures.DOCX]

**Supplementary Information**

**Mobile genetic elements drive antimicrobial resistance gene spread in *Pasteurellaceae* species**

Giarlã Cunha da Silva^1, #^, Osiel Silva Gonçalves^2, #^, Jéssica Nogueira Rosa^1^, Kiara Campos França^2^, Paul Richard Langford^3^, Janine Thérèse Bossé^3, *^, Mateus Ferreira Santana^2, *^, Denise Mara Soares Bazzolli^1, *^

^1^Laboratório de Genética Molecular de Bactérias, Departamento de Microbiologia, Instituto de Biotecnologia Aplicada à Agropecuária, Universidade Federal de Viçosa, Viçosa, Minas Gerais, Brazil.

^2^Grupo de Genômica Evolutiva Microbiana, Laboratório de Genética Molecular de Microrganismos, Instituto de Biotecnologia Aplicada à Agropecuária, Universidade Federal de Viçosa, Minas Gerais, Brazil.

^3^Section of Paediatrics, Department of Medicine, Imperial College London, London, United Kingdom.

^*^ Corresponding authors:

dbazzolli@ufv.br; +55 31 3612 2454

j.bosse@imperial.ac.uk; +44 (0)20 7594 1803

mateus.santana@ufv.br; +55 31 3612 2452

^#^ These authors contributed equally to this work


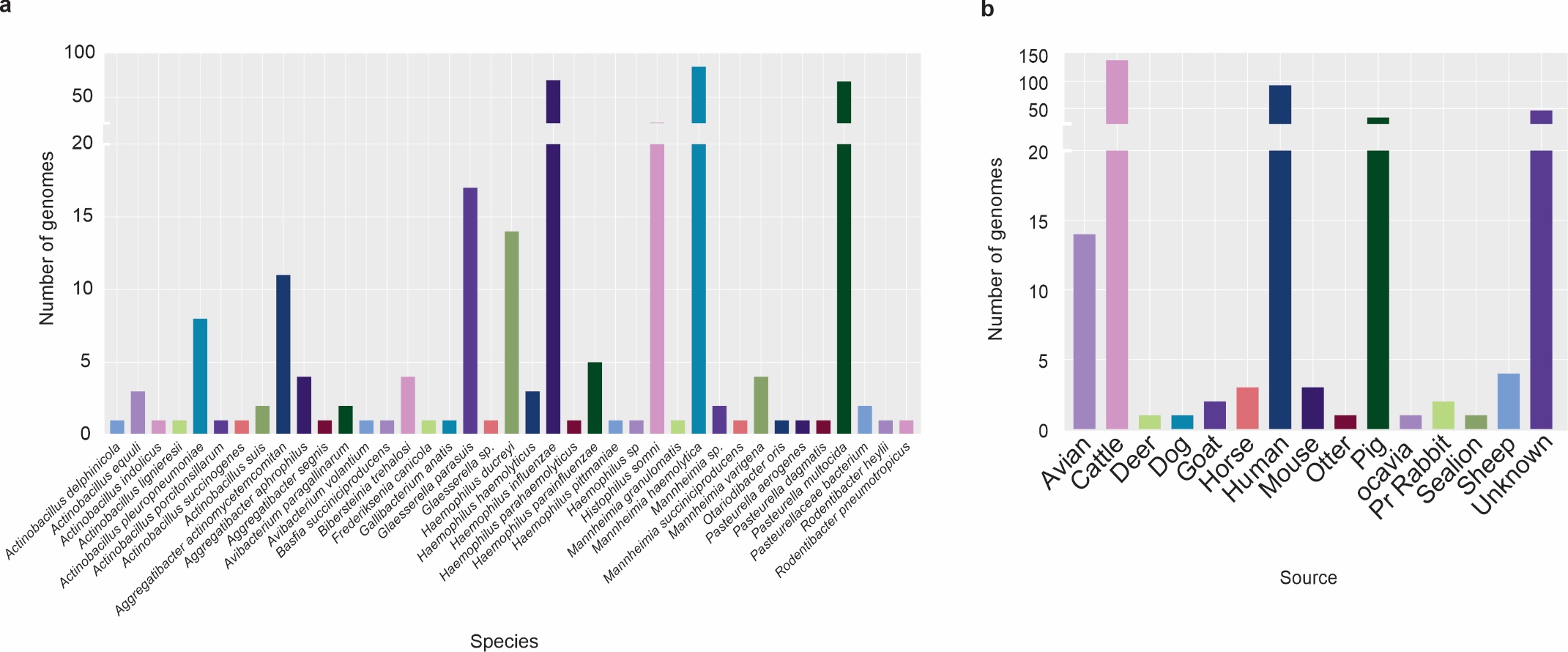


**Supplementary Fig. 1. General information regarding the genome’s dataset. a**, The Bar graph of *Pasteurellaceae* genomes distribution in a wide number of species in the dataset. **b,** The Bar graph of species from different sources including human and distinct animal sources.


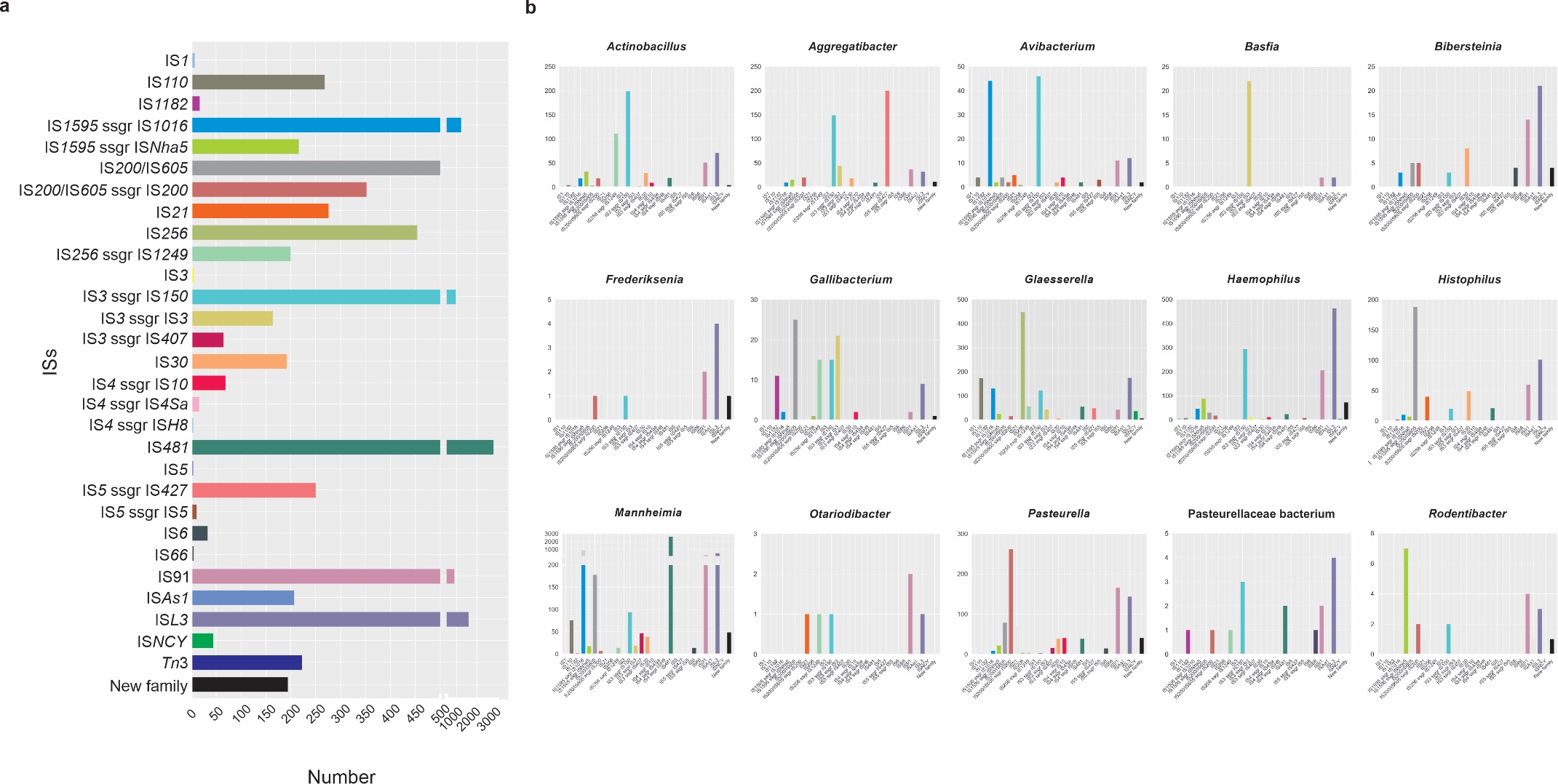
 **Supplementary Fig. 2. Comparison of IS elements against the dataset. a**, The total values of IS families mapped in our dataset, which indicates the wide variety of IS associated with the genomes**. b,** The distribution of ISs families throughout the *Pasteurellaceae* genera. The elements classified as "new family" by ISsaga have not been further investigated.


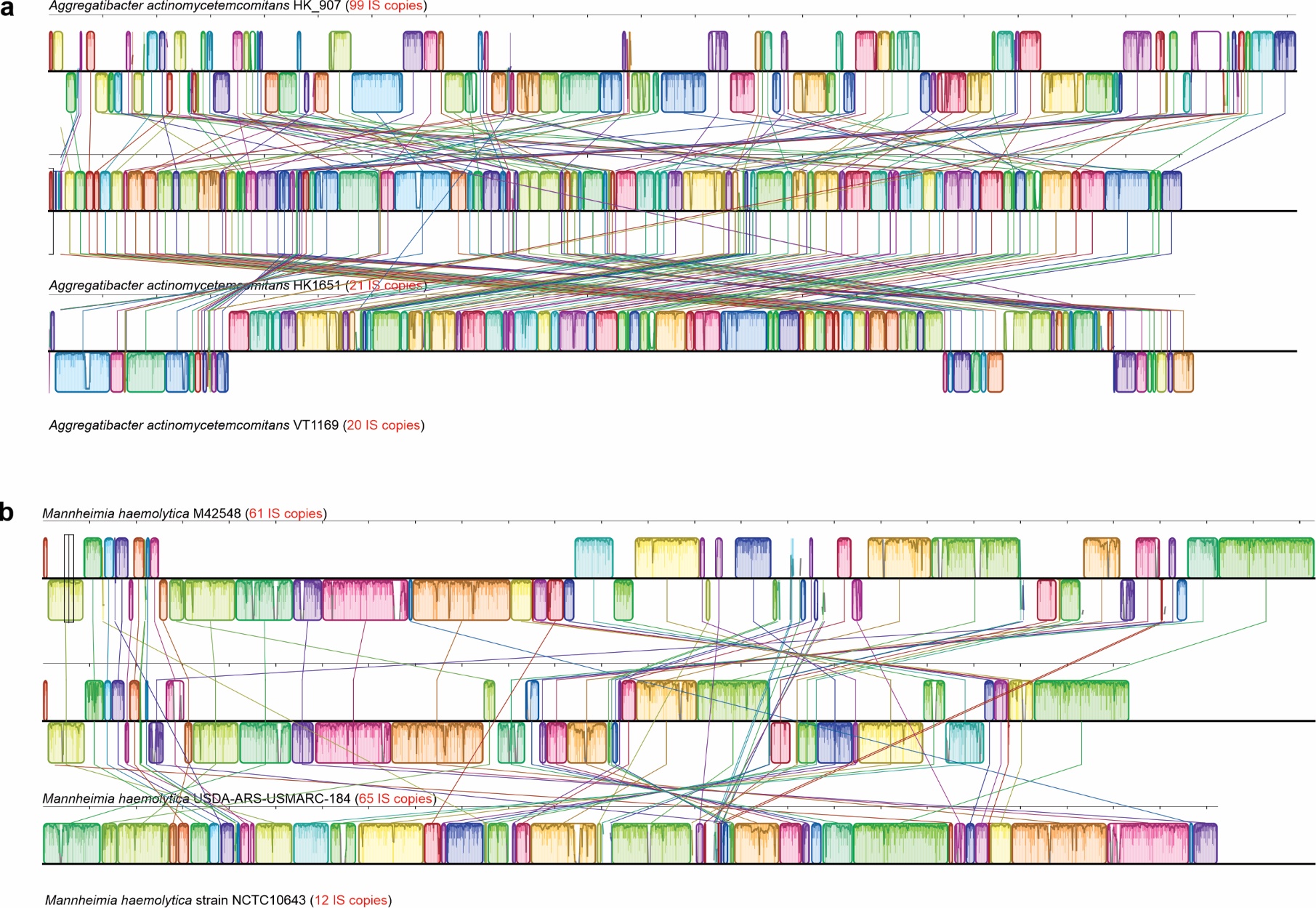


**Supplementary Fig. 3. The impact of ISs in the organization of the genome of *Pasteurellaceae* members.** Synteny analysis shows numerous internal rearrangements in the whole genome sequences of *A*. *actinomycetemcomitans* (**a**), and *M*. *haemolytica* (**b**). Colored blocks represent co-linear blocks, and the number of IS elements found in the genome is shown in red write.


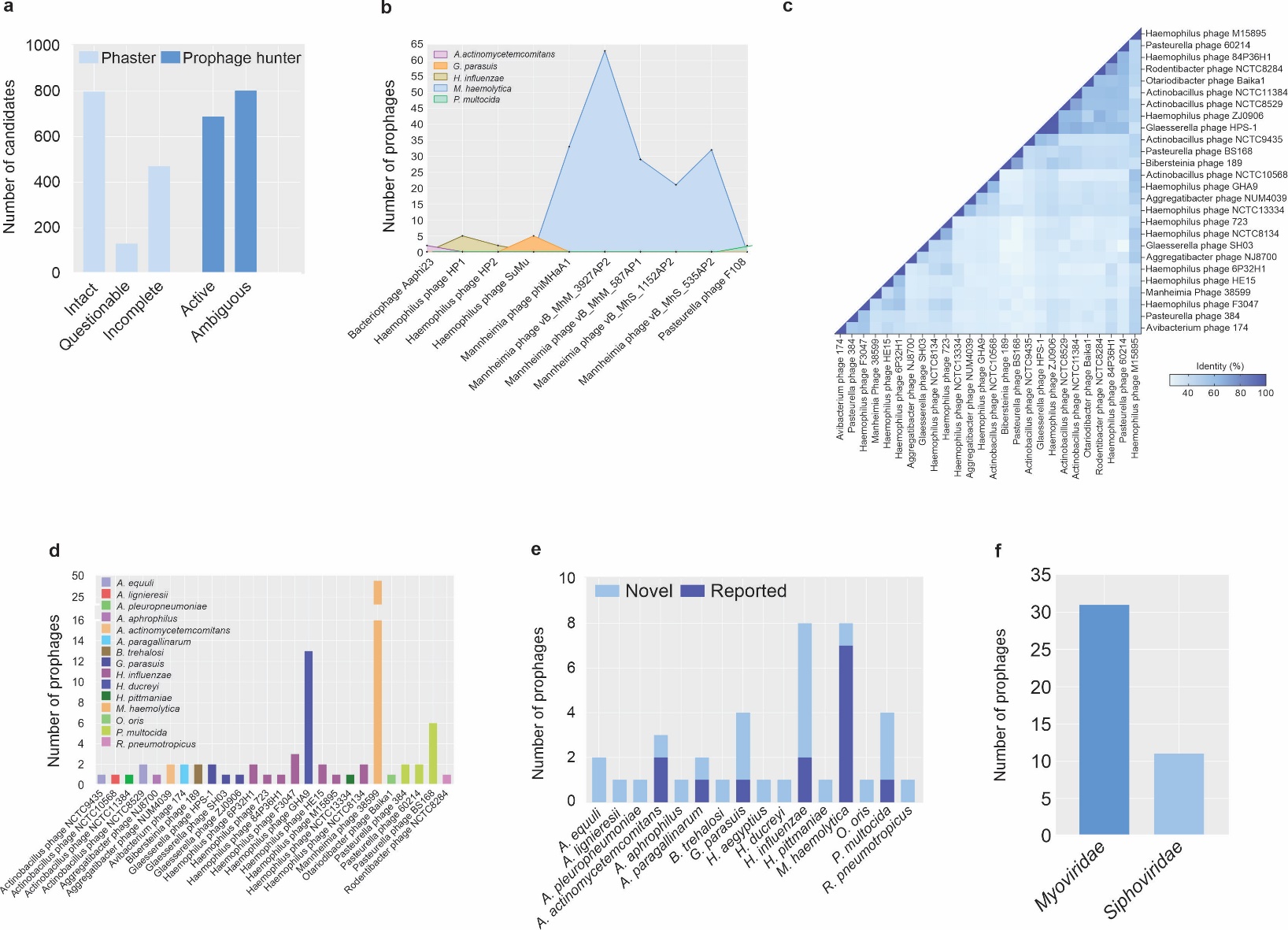


**Supplementary Fig. 4. Prophages of *Pasteurellaceae* are disseminated among species. a**, Results of prophage-like prediction from PHASTER and Prophage hunter predictors with their respective classes of candidates for each software. **b**, Identification of reported prophages of the family in the dataset genomes. **c**, Heatmap based on matrix identity of the novel prophages here described. **d**, Number of the novel prophages described in this study among the dataset genomes. **e**, The comparison of novel and reported prophages of the *Pasteurellaceae* species. **f,** The distribution of viral family from the novel and reported prophage to the *Pasteurellaceae*.


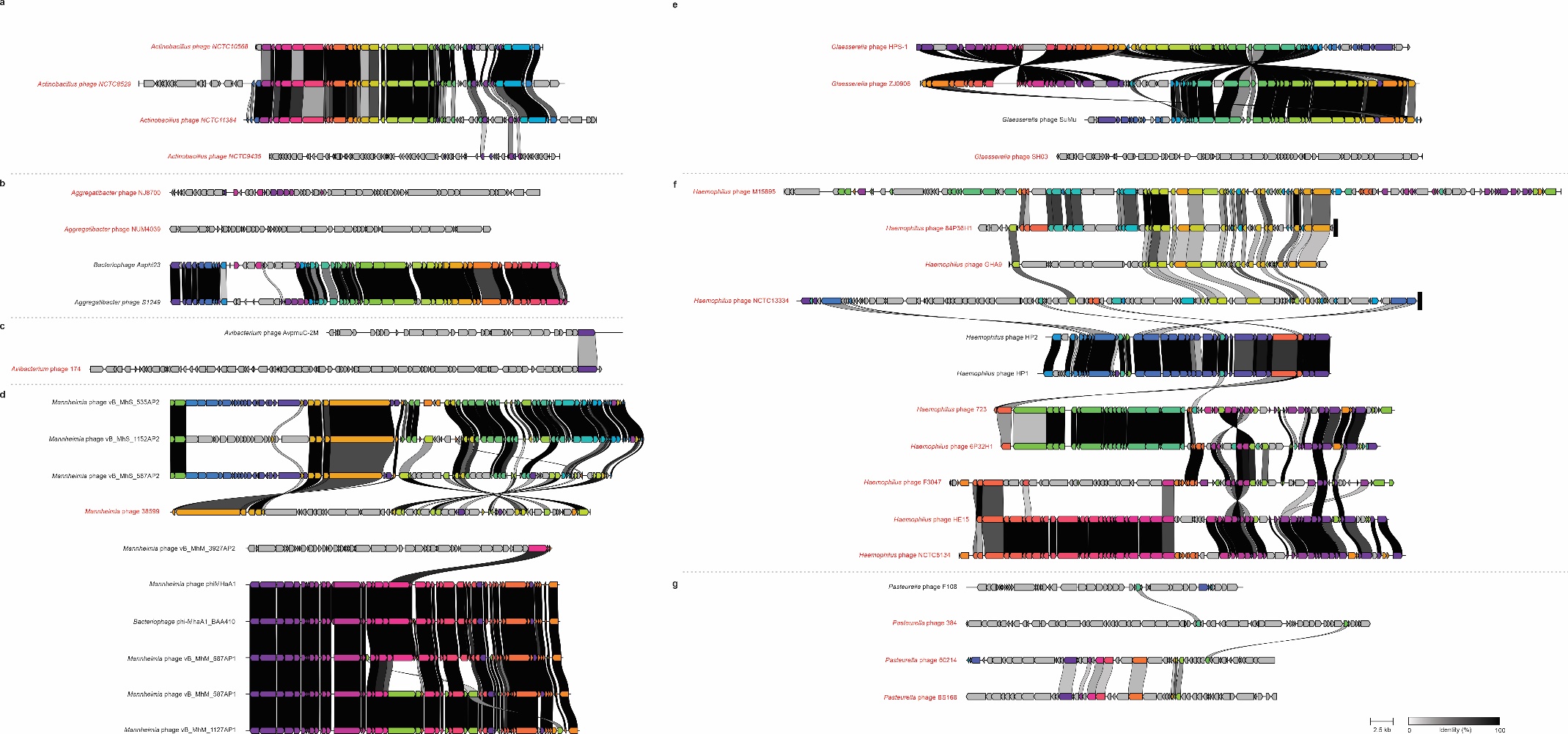
**Supplementary Fig. 5. Synteny analysis for the sequence comparison of novel prophages and the prophages reported for the family.** Synteny analysis generated using clinker and clustermap.js of prophages grouped by genera (**a**, *Actinobacillus*; **b**, *Aggregatibacter*; **c**, *Avibacterium*; **d**, *Mannheimia*; **e**, *Glaesserella*; **f**, *Haemophilus*; **g**, *Pasteurella*) shows the novel prophages (in red) are different from those already reported for the family.


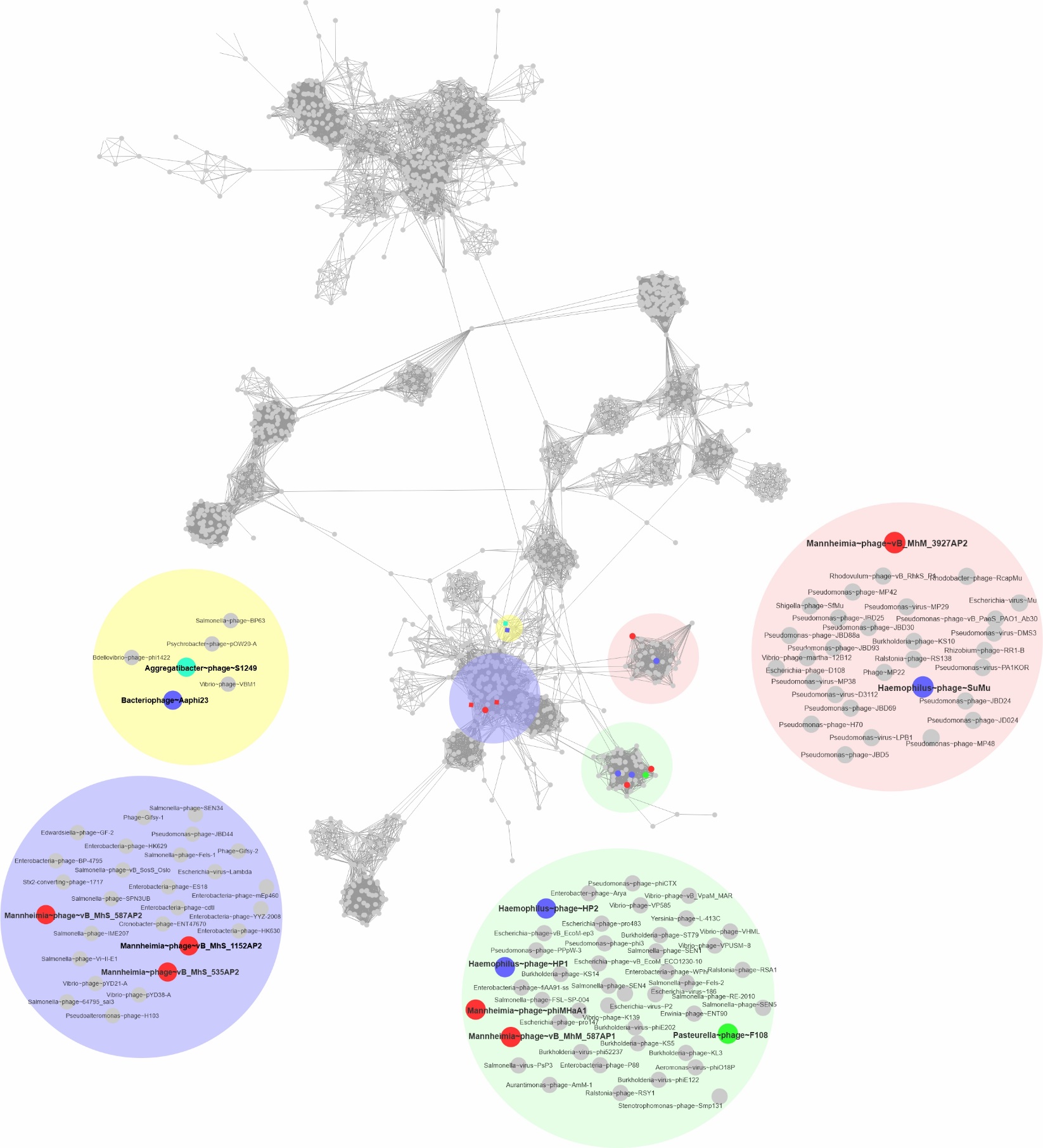


**Supplementary Fig. 6. Bipartite network analysis of reference viral species for Pasteurellaceae using vConTACT. Color dots indicate the viral genome from the family**. Each node represents a viral genome from RefSeq and edges between nodes indicate a statistically significant relationship between the protein profiles of their viral genomes.


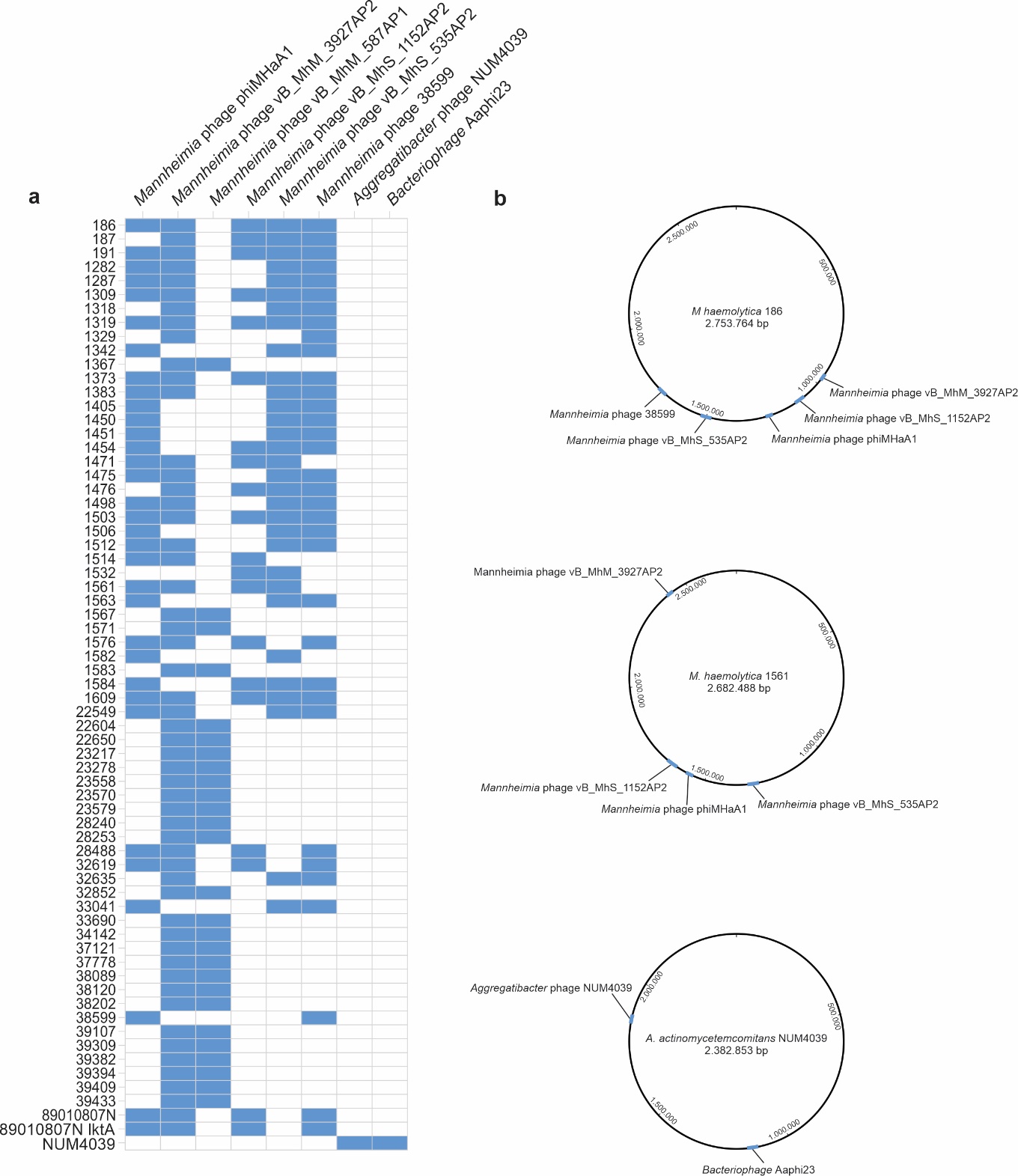


**Supplementary Fig. 7. The analysis of poly-lysogeny in the genomes of *Pasteurellaceae*. a**, The presence of multiple prophage elements marked in blue boxes in the genomes of *M. haemolytica* and *A. actinomycetemcomitans* (strain NUM4039). The bacterial strains are shown on the left side. **b**, Poly-lysogeny is represented in some genomes of *M. haemolytica* and *A. actinomycetemcomitans.* This figure shows the multiple prophages with respective localization in the strain’s genomes.


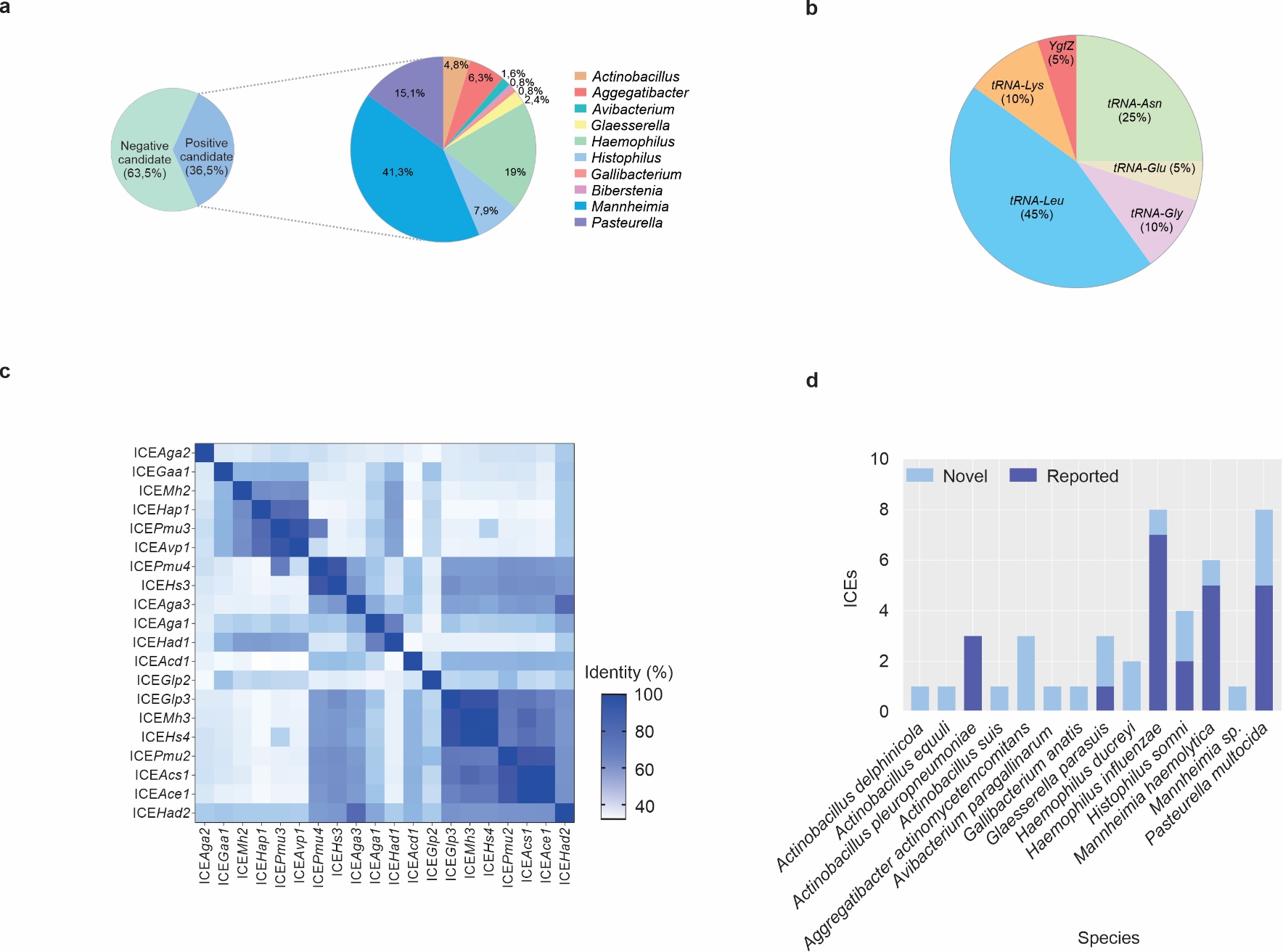


**Supplementary Fig. 8. Overview of novel ICEs. a**, Results from OriTFinder showed regions with high potential for self-transferability in 126 genomes of the dataset comprising different genera. **b**, Different tRNAs as integration site. **c**, Heatmap based on matrix identity of the novel ICEs described in this study. **d**, The distribution of novel and reported ICEs of the *Pasteurellaceae* among bacterial species.


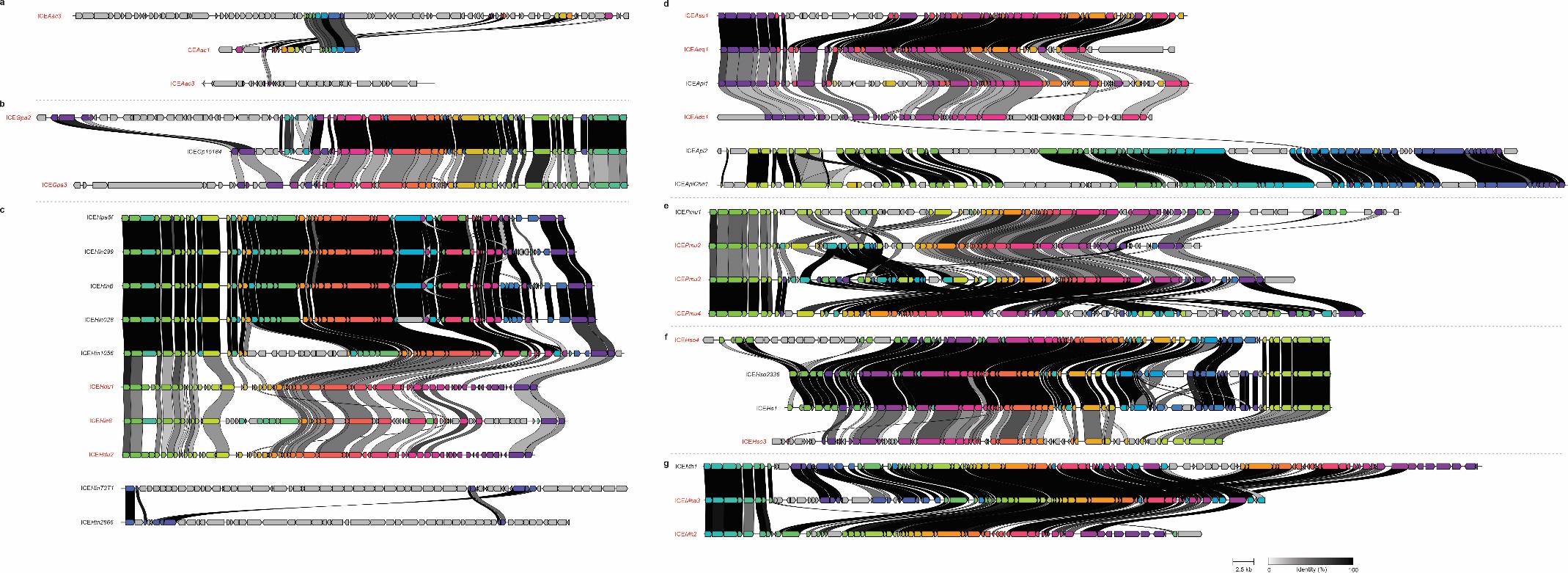


**Supplementary Fig. 9. Synteny analysis for the sequence comparison between novel ICEs and reported for the family.** Synteny analysis generated using clinker and clustermap.js of ICEs showing the difference of structure for each element grouped by genera (**a**, *Aggregatibacter*; **b**, *Glaesserella*; **c**, *Haemophilus*; **d**, *Actinobacillus*; **e**, *Pasteurella*; **f**, *Histophilus*; **g**, *Mannheimia*) shows the novel ICEs (in red) are different from those already reported for the family.


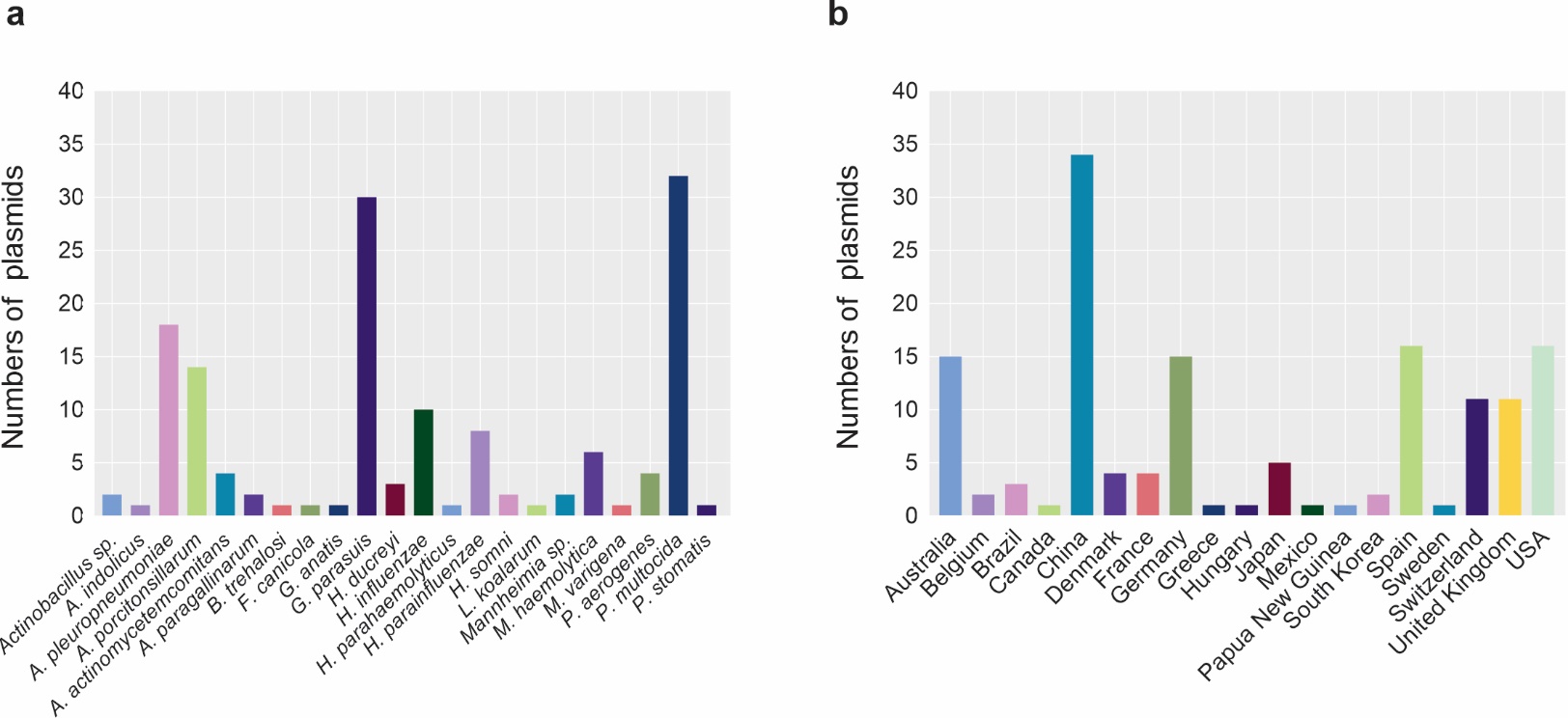


**Supplementary Fig. 10. General information regarding the plasmid dataset. a**, The distribution of *Pasteurellaceae* plasmids in a wide number of species. **b,** Graph bar of the plasmids from different countries.


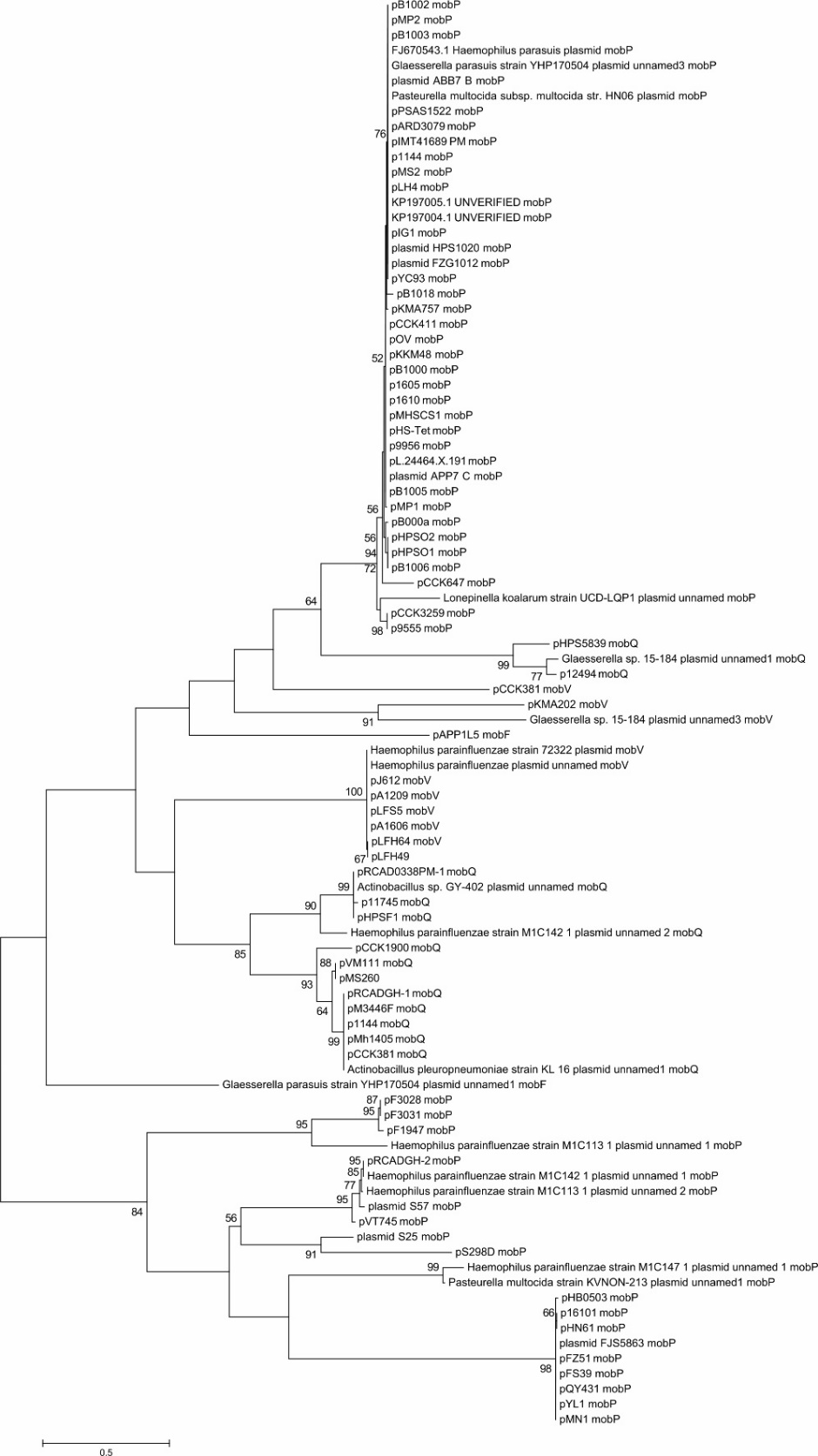


**Supplementary Fig. 11. Full phylogenetic tree of MOB genes.** The evolutionary history of MOB gene from plasmids inferred by using the Maximum Likelihood method based on the General Time Reversible model was conducted in MEGA X. The tree is drawn to scale, with branch lengths in the same units as those of the evolutionary distances used to infer the phylogenetic tree.


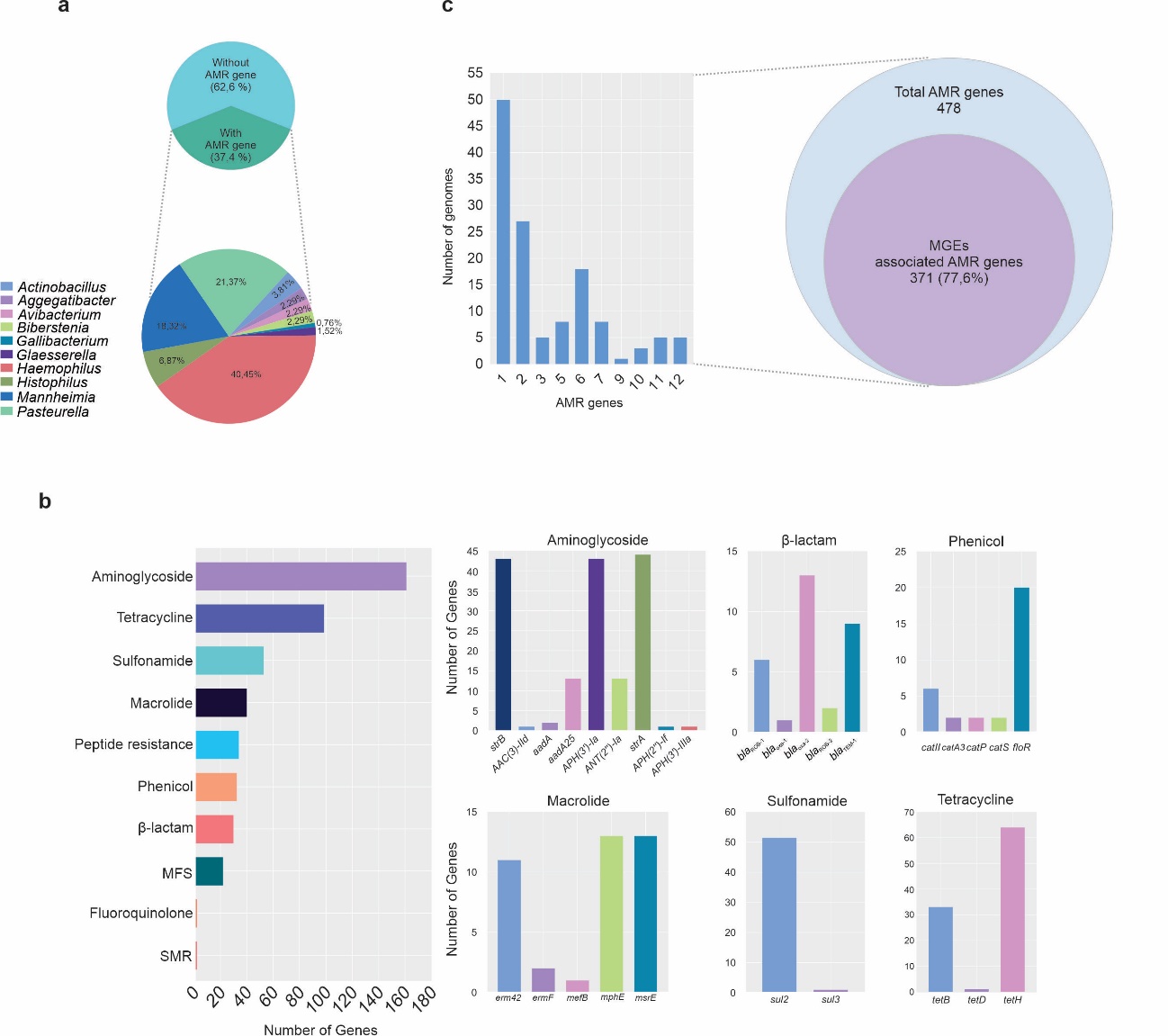


**Supplementary Fig. 12. Overview of AMR genes and their classes identified in the genomes.** **a**, Result of AMR prediction showed that these genes were found in 131 genomes, which are distribute among 10 genera. **b**, Number of AMR genes per class found associated with genomes. **c**, Number of AMR genes found within genomes and localization of AMR identified.


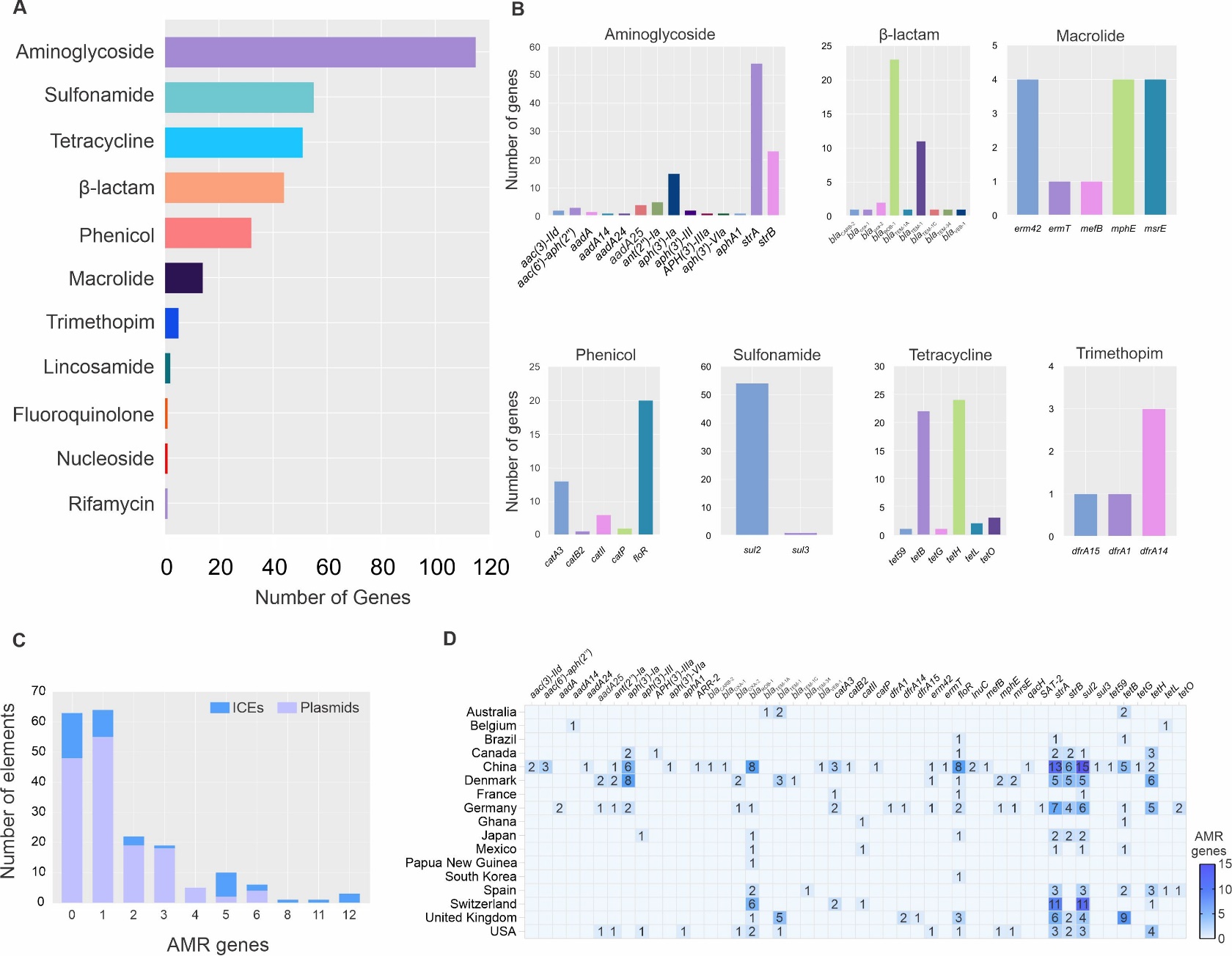


**Supplementary Fig. 13. Overview of AMR genes and their classes identified in the MGEs. a**, The distribution by classes of AMR genes associated with MGEs in *Pasteurellaceae*. **b**, The number of AMR genes per class found associated with MGEs. **c**, Number of AMR genes found within MGEs (Plasmids and ICEs). **d**, Distribution of AMR genes associated with MGEs per country. The values are indicated within the square.
